# Parathyroidectomy and survival in a cohort of Italian dialysis patients: results of a multicenter, observational, prospective study

**DOI:** 10.1007/s40620-023-01658-0

**Published:** 2023-06-23

**Authors:** Lida Tartaglione, Silverio Rotondi, Filippo Aucella, Mario Bonomini, Maria Rosa Caruso, Francesco Casino, Carlo Cuzziol, Alessio Farcomeni, Armando Filippini, Carlo Lomonte, Rocco Marinelli, Davide Rolla, Filomena Rubino, Giuseppe Seminara, Marzia Pasquali, Sandro Mazzaferro, on behalf of The Italian Study Group on Mineral Metabolism of the Italian Society of Nephrology, C. Bagnis, S. Bisegna, F. Conte, C. Camerini, M. R. Caruso, P. Messa, E. Corghi, M. Farina, A. Galassi, F. Malberti, P. Poiatti, P. Ruggiero, G. Vezzoli, M. Cozzolino, M. Nordio, G. Meneghel, N. Chiappini, A. Icardi, M. Nordio, D. Rolla, C. Grimaldi, G. Cianciolo, D. Casu, D. Pilloni, M. Scrivano, G. Sini, M. A. Sechi, B. Contu, S. Grussu, L. Gazzanelli, A. Pani, G. Brotzu, M. Bonomini, M. Polidoro, L. Onorato, M. Leonardi, F. Amoroso, M. Baldinelli, M. Morosetti, E. Boccia, S. Chicca, P. Martina, A. Di Silva, D. A. Ordonez, A. Filippini, A. Marinelli, C. Massimetti, P. Menè, I. Napoletano, P. Sfregola, E. Retico, F. Bondatti, F. Cannula, F. Rubino, C. Cuzziol, C. Di Cicco, A. Flammini, D. Mantella, R. Nacca, F. Violi, G. Pulcinelli, A. Balducci, S. Baldini, M. Martello, M. Di Luca, M. Brigante, E. Nunzi, F. Aucella, C. Lo Monte, P. Magarelli, F. Casino, L. D’Apice, L. Morrone, G. G. Battaglia, V. Savica, R. Roberti

**Affiliations:** 1 Nephrology Unit, Azienda Policlinico Umberto I, Rome, Italy; 2 Department of Translational and Precision Medicine, Sapienza University of Rome, Viale del Policlinico 155, 00161 Rome, Italy; 3 Nephrology Unit, Casa Sollievo della Sofferenza, Monte Rotondo, Italy; 4 Department of Medicine and Aging Sciences, G. D’annunzio University, Chieti, Italy; 5 Papa Giovanni XXIII Hospital, Bergamo, Italy; 6 Nephrology Unit, Matera, Italy; 7 Nephrology Unit ARS Medica Rome, Rome, Italy; 8 Department of Economics and Finance, Tor Vergata University Rome, Rome, Italy; 9 Casilino Hospital Rome, Rome, Italy; 10 Nephrology Department, Ospedale Generale Regionale “F. Miulli”, Acquaviva delle Fonti, Italy; 11 Nephrology Unit, Madonna della Fiducia, Rome, Italy; 12 Sant’Andrea Hospital La Spezia, La Spezia, Italy; 13 Nephrology Unit Sora Hospital, Sora, Italy; 14 Agrigento Hospital Agrigento, Agrigento, Italy

**Keywords:** Parathyroidectomy, Hemodialysis, Mortality, CKD-MBD

## Abstract

**Background:**

Severe secondary hyperparathyroidism (SHPT) is associated with mortality in end stage kidney disease (ESKD). Parathyroidectomy (PTX) becomes necessary when medical therapy fails, thus highlighting the interest to compare biochemical and clinical outcomes of patients receiving either medical treatment or surgery.

**Methods:**

We aimed to compare overall survival and biochemical control of hemodialysis patients with severe hyperparathyroidism, treated by surgery or medical therapy followed-up for 36 months. Inclusion criteria were age older than 18 years, renal failure requiring dialysis treatment (hemodialysis or peritoneal dialysis) and ability to sign the consent form. A control group of 418 patients treated in the same centers, who did not undergo parathyroidectomy was selected after matching for age, sex, and dialysis vintage.

**Results:**

From 82 Dialysis units in Italy, we prospectively collected data of 257 prevalent patients who underwent parathyroidectomy (age 58.2 ± 12.8 years; M/F: 44%/56%, dialysis vintage: 15.5 ± 8.4 years) and of 418 control patients who did not undergo parathyroidectomy (age 60.3 ± 14.4 years; M/F 44%/56%; dialysis vintage 11.2 ± 7.6 y). The survival rate was higher in the group that underwent parathyroidectomy (Kaplan–Meier log rank test = 0.002). Univariable analysis (HR 0.556, CI: 0.387–0.800, *p* = 0.002) and multivariable analysis (HR 0.671, CI:0.465–0.970, *p* = 0.034), identified parathyroidectomy as a protective factor of overall survival. The prevalence of patients at KDOQI targets for PTH was lower in patients who underwent parathyroidectomy compared to controls (PTX vs non-PTX: PTH < 150 pg/ml: 59% vs 21%, *p* = 0.001; PTH at target: 18% vs 37% *p* = 0.001; PTH > 300 pg/ml 23% vs 42% *p* = 0.001). The control group received more intensive medical treatment with higher prevalence of vitamin D (65% vs 41%, *p* = 0.0001), calcimimetics (34% vs 14%, *p* = 0.0001) and phosphate binders (77% vs 66%, *p* = 0.002).

**Conclusions:**

Our data suggest that parathyroidectomy is associated with survival rate at 36 months, independently of biochemical control. Lower exposure to high PTH levels could represent an advantage in the long term.

**Graphical abstract:**

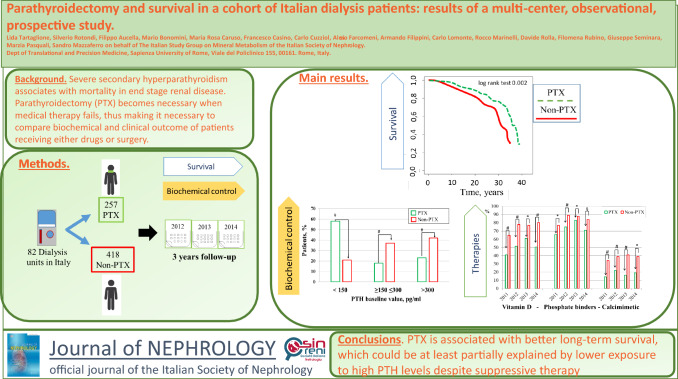

**Supplementary Information:**

The online version contains supplementary material available at 10.1007/s40620-023-01658-0.

## Introduction

Secondary hyperparathyroidism (SHPT) in end-stage renal disease (ESRD) is associated with disturbances in mineral metabolism, metabolic bone disease and renal osteodystrophy, bone fractures, vascular calcifications [1–4] and the eventual increase of cardiovascular disease and mortality. Conventional treatment of SHPT with phosphate binders, vitamin D receptor activators (VDRAs) and calcimimetics [5–7] may not allow adequate biochemical control, and parathyroidectomy (PTX) is still recommended in severe cases failing to respond to medical therapy [8].

Parathyroidectomy rapidly lowers parathyroid hormone (PTH) serum levels with improvement of serum calcium and phosphate control, and has potentially favorable effects on cardiovascular survival. Indeed, a lower risk of mortality is reported when all three standard biochemical indicators of metabolic control (namely Ca, P and PTH) reach the target levels recommended by K-DOQI at least once [9]. However, targeting all three biomarkers is not easily accomplished after PTX [10–12]. In fact, in the long term after surgery, hypoparathyroidism is frequent and both low and high levels of PTH are associated with increased cardiovascular morbidity and mortality, in a typically U-shaped modality [13]. Notwithstanding, available observational studies in hemodialysis (HD) patients describe reduced all-cause and cardiovascular mortality rates after PTX in the long term[14–17], apparently regardless of sub-optimal biochemical control. The pathophysiological link between PTX and improved survival is not clear but may include the reported effects of PTH on left ventricular hypertrophy, blood pressure control, erythropoietin-resistant anemia, nutritional status and humoral and/or cellular immunity, independently of calcium and phosphate control and of prescribed specific therapies [18–21]. Regrettably, prospective, randomized-controlled trials comparing the mortality rates of HD patients receiving either medical or surgical therapy for severe SHPT are not available, and will never be carried out due to ethical issues [22]. Therefore, observational studies, despite suffering selection bias are still the main source of data that provide information on the relationship between PTX, biochemical control and mortality rates in HD patients. This paper reports the results of a multicenter, observational, prospective cohort study aimed at evaluating the impact of PTX on survival in an Italian cohort of HD patients.

## Methods

### Study population and data collection

In this paper, we report the prospective, observational part of a multicenter cohort study on PTX that involved 149 Italian dialysis Units, whose protocol was approved by the Ethics Committee of the Policlinico Umberto I in Rome (prot. N° 888/09), and whose baseline data have already been published [12]. Briefly, in this study, inclusion criteria were age older than 18 years, renal failure requiring dialysis treatment (hemodialysis or peritoneal dialysis) and ability to sign the consent form. Data from each Unit of the 528 enrolled patients with PTX history were provided by a referent physician who recorded medical history, timing of PTX, type of surgery, laboratory data, and prescribed SHPT medications in a dedicated data sheet. In addition, information about sex, age and dialysis vintage of all 12,515 patients receiving treatment in the involved Units provided a population from which a control group could be selected.

### Follow-up data

**Fig. 1:**
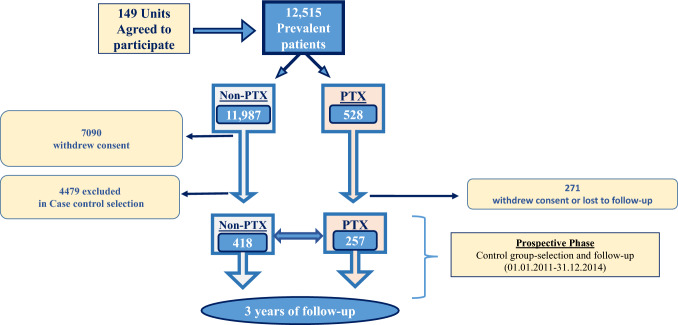
Flowchart for the study population. Abbreviations: *PTX* parathyroidectomy; *PTH* Parathyroid hormone; *Ca* calcium; *P* phosphate

Further to the baseline descriptive phase, the protocol also included a prospective observational follow-up, lasting three years, which, however, did not include 67 units. Thus, as schematically reported in Fig. 1, for the follow up phase of the study (the results of which are reported in this paper) we had 257 PTX patients and 4897 controls, among whom we selected, in 2011, 418 non-PTX cases that were similar in terms of age, sex, and dialysis vintage to the study group. Clinical and therapeutic updates were then collected prospectively for the selected patients for three consecutive years (from 01.01.2012 to 31.12.2014). We recorded fatal events from any cause, prescribed medications for SHPT control (vitamin D and calcium-based therapies, calcimimetics, phosphate binders) and laboratory data pertinent to mineral metabolism (PTH, calcium and phosphate) during the three years of follow-up.

The study complied with the ethical standards of the institutional and/or national research committee and with the 1964 Helsinki declaration and its later amendments or comparable ethical standards.

### Endpoints

The primary endpoint was the overall survival rate of PTX and non-PTX patients during the 36 months of follow-up.

The secondary endpoint was the prevalence of patients reaching biochemical targets for mineral metabolism, as defined by the K-DOQI ranges for Ca, P and PTH [9], in the two groups of PTX and non-PTX patients.

### Statistical analysis

Data are expressed as mean ± SD for variables with a Gaussian distribution or median [25–75th percentiles] when the distribution was non Gaussian. We used the Kolmogorov–Smirnov test to evaluate normality of continuous measurements. Parametric tests, chi squared test for qualitative and t-test for quantitative variables, were used to compare measurements between the groups. When the normality assumption was not tenable, Mann–Whitney was used to test for significant differences. All tests were two-tailed and (adjusted) *P*-values < 0.05 were considered as statistically significant. When general r-by-c contingency tables yielded statistical significance, we proceeded to the evaluation of two-by-two sub-tables of interest. In that case, significance levels were Bonferroni-adjusted by multiplication by the number of two-by-two tables evaluated. The family-wise significance level was fixed at 5%, so that a Bonferroni adjusted *p* value below 0.05 was considered as statistically significant after taking into account multiplicity. The time-to-event outcomes association in PTX and non-PTX patients was evaluated starting from the date of hemodialysis inception, through stratified Kaplan–Meier curves and associated log-rank tests and/or univariable Cox regression models. As multivariable analyses, we used Cox regression models, where the final set of predictors was selected by means of forward selection based on Akaike Information Criterion. We further evaluated the effect of PTX through a propensity-score matched analysis. First we estimated the probability of obtaining the treatment based on gender, age, diabetes, albumin and hemoglobin levels. One-to-one matching was then performed based on the estimated propensity score, and the matched subset was used in a Cox regression model for estimation of the Average Treatment effect for the Treated (under selection-on-observables assumptions). Balance was evaluated through Standardized Mean Differences (SMD), where an SMD < 10% indicated a good balance.

Analyses were performed using the open source software package *R* version 4.2.1.

## Results

### Patient characteristics

Table 1 describes the main clinical and biochemical characteristics of the PTX and non-PTX groups which were similar with regard to age and sex distribution, but different concerning dialysis vintage (PTX = 15.5 ± 8.4 *vs* non-PTX = 11.2 ± 7.6 years, *p* < 0.0001) (Table 1). Patients in the PTX group, who underwent surgery on average 8 years after dialysis inception, showed a higher prevalence of glomerular diseases and tubulointerstitial nephropathies, as compared to the non-PTX group (Table 1). History of comorbidities did not differ between the two groups, in particular regarding the incidence of cardiovascular diseases (peripheral vascular disease, ischemic heart disease, and/or heart failure). In addition, no difference was observed in the prevalence of arterial hypertension (identified as current antihypertensive drug prescription), while diabetes was less frequently in PTX patients (6% *vs* 14%, *p* = 0.002) (Table 1).

**Table 1:** Baseline characteristics in PTX and non-PTX patients

	PTX	Non-PTX	*P*
Total Patients	257	418	
Age, years	58.2 ± 12.8	60.3 ± 14.4	0.057
Dialysis vintage, y	15.5 ± 8.4	11.2 ± 7.6	< .0001
Female, %	56	56	1
Male, %	44	44	1
Causes of ESRD ( %)			
Glomerular diseases	43	30	< .0001
Tubulointerstitial nephropathies	12	6.4	< .0001
Nephroangiosclerosis	10	15	0.025
ADPKD	10	13.8	0.07
Uncertain ESRD etiology	25	34.8	0.001
Comorbidities (%)			
Arterial hypertension	44	46	0.624
Diabetes	6	14	0.002
Peripheral vascular disease,	13	12	0.852
Ischemic heart disease	14	12	0.246
Heart failure	7	9	0.545
Dyslipidemia	32	30	0.644
Laboratory tests			
Ca, mg/dl	8.79 ± 0.67	9.04 ± 0.68	< .0001
Phosphate, mg/dl	4.98 ± 1.42	5.11 ± 1.34	0.294
PTH, pg/ml	102.0 (17.2–337.15)	250.0 (163.0–400)	< .0001
Albumin, gr/dl	3.85 ± 0.52	3.69 ± 0.45	< .0001
Hemoglobin, gr/dl	11.5 ± 1.0	11.6 ± 0.8	0.651

Data are shown as mean ± standard deviation, median (IQR) or percentage. Chi-squared test was used to test for any significant differences between qualitative variables. *T*-test or Mann–Whitney were used to test for any significant differences between quantitative variables

Abbreviations: *D* dialysis; *PTX* Parathyroidectomy; *ESRD* end stage renal disease; *Ca* calcium; *P* phosphate; *PTH* parathyroid hormone, *ADPKD* autosomal dominant polycystic kidney

### Survival analysis

**Fig. 2:**
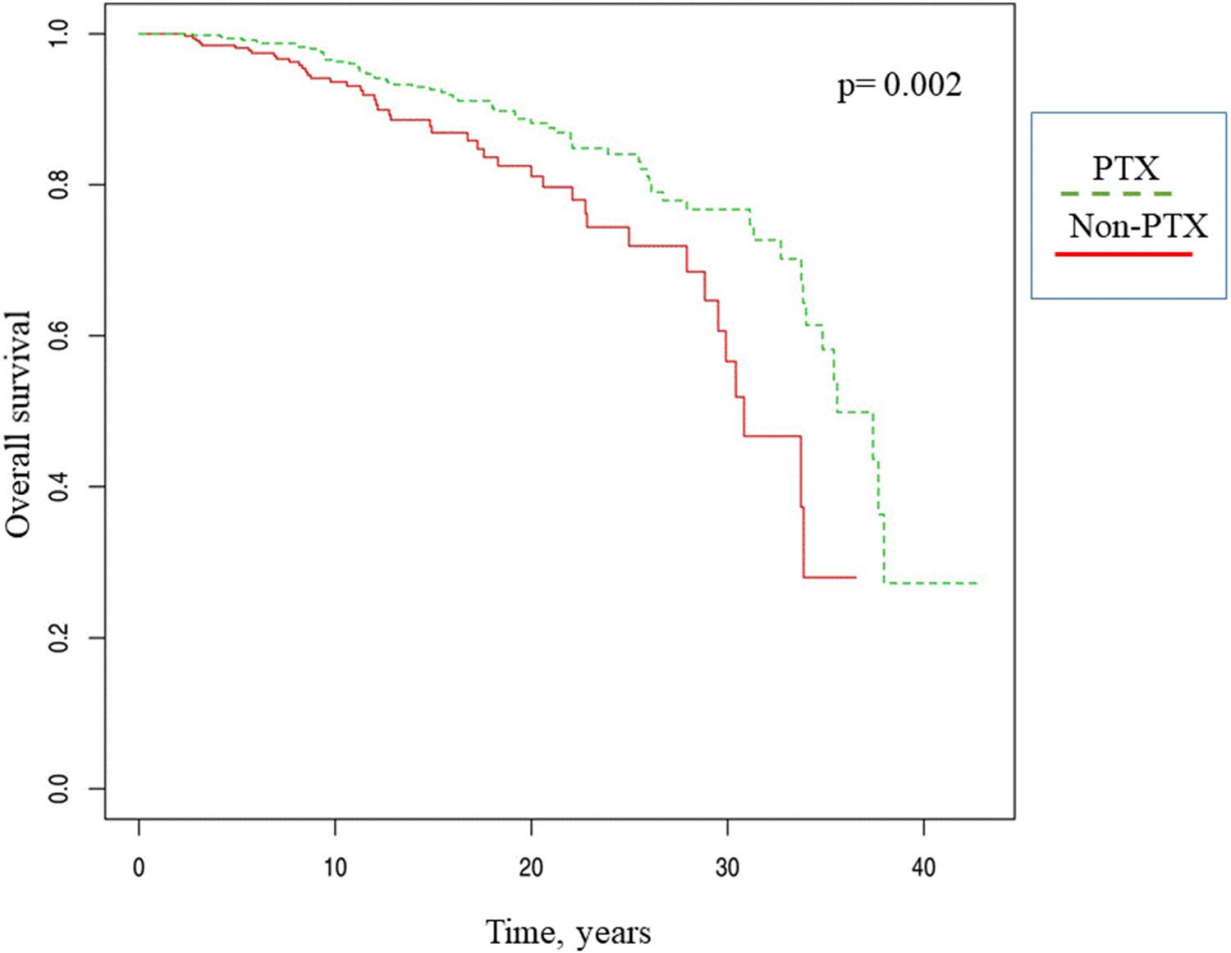
Overall survival, PTX vs. Non-PTX. Time 0 was the date of starting hemodialysis treatment. Kaplan–Meier log rank test = 0.002

The survival curves in the two groups of patients, evaluated from the date of hemodialysis inception, clearly show the long-term lower mortality rate of the PTX group (Kaplan–Meier log rank test = 0.002 Fig. 2). This result was also confirmed when the survival curves were considered from the beginning of the three years of follow-up (Kaplan–Meier log rank test = 0.023, Fig. 1 supplemental material).

### Biochemical control and effect of therapies

**Fig. 3:**
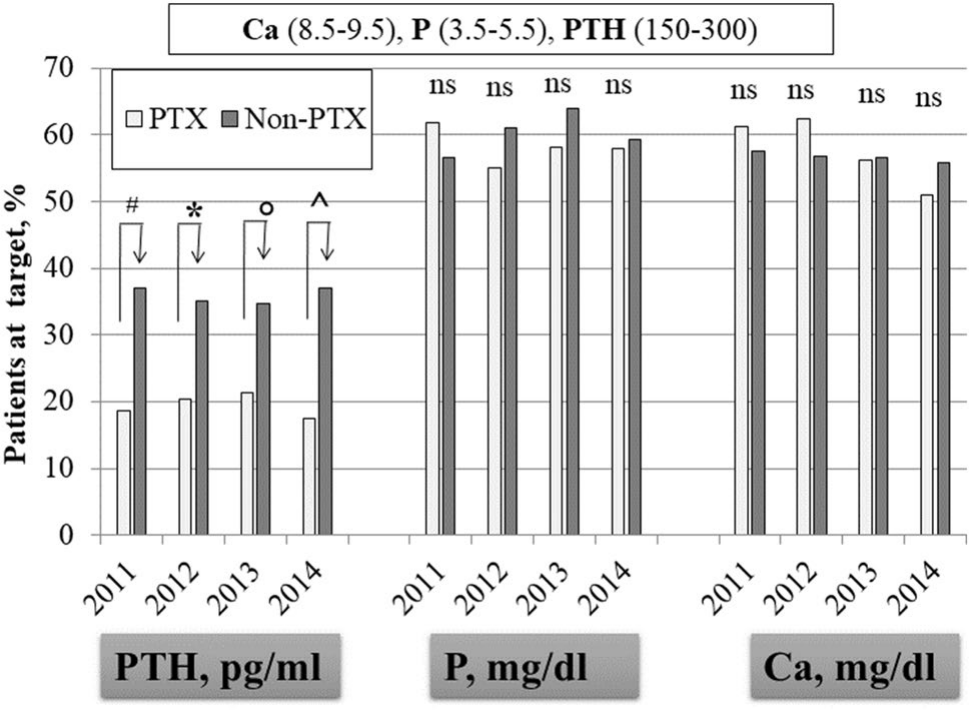
Baseline percentage of patients distributed according to PTH K-DOQI target values.^#^PTX vs Non-PTX X^2^, *p* = 0.0001. Abbreviation: Parathyroid hormone

**Fig. 4:**
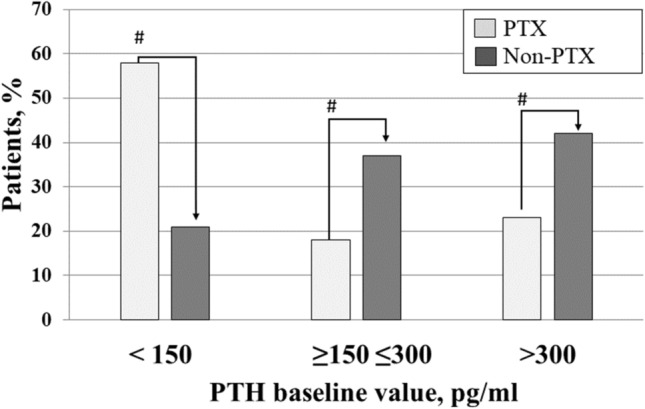
Patients at K-DOQI targets for calcium, phosphate and PTH during follow-up #PTX vs Non-PTX X2 *p* = 0.0001 *PTX vs Non-PTX X2 *p* = 0.0002; °PTX vs Non-PTX X2 *p* = 0.0025; ^PTX vs Non-PTX X2 *p* = 0.0003. Abbreviation: *PTH* Parathyroid hormone; *Ca* calcium; *P* phosphate

**Fig. 5:**
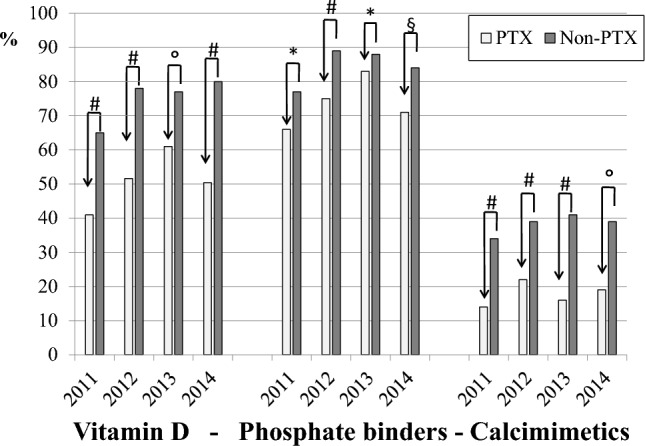
Therapies during follow-up. Vitamin D includes calcitriol, intravenous Vitamin D Receptor Activators and other forms. Phosphate binders include both calcium and non calcium based binders. #X2 *p*<0.0001; * *X*2 *p*= 0.002; *X*2 *p* = 0.0005; $ X2 *p*= 0.01

Serum calcium (8.79 ± 0.67 *vs* 9.04 ± 0.68 mg/dl, *p* < 0.0001) and intact PTH (102.0, IQR: 17.2–337.1 *vs* 250, IQR: 163–400 pg/ml, *p* < 0.0001) were significantly lower in the PTX compared to the non-PTX group of patients, while phosphate was not different (Table 1). We then compared biochemical values, therapies and any-cause mortality in the two groups during the three consecutive years of follow-up. During the observation period, fifty-four patients (21%) in the PTX group and 181 (43%) in the non-PTX group were lost to follow-up, leaving 203 and 237 cases, respectively, for comparison. As illustrated in Fig. 3, at baseline, the prevalence of cases below, at target or above the PTH KDOQI targets was different between the two groups. In particular the percentage of patients below 150 was higher in the PTX group (59 vs 21%, *p* = 0.001), while the percentage at target or above was significantly higher in the non-PTX group (18 vs 37%; *p* < 0.0001and 23 vs 42%, *p* = 0.0001, respectively). Notably, during follow-up, these differences were systematically confirmed (Fig. 4). No difference was evident for serum calcium and phosphate (Fig. 4). As for SHPT therapies, the non-PTX group received more aggressive treatment characterized by significantly higher prescriptions of vitamin D, phosphate binders and calcimimetics (Fig. 5). Moreover, PTX patients received more calcitriol and calcium-based phosphate binders than the control group, which, by comparison, received more vitamin D receptor activators and non-calcium-based phosphate binders (Table 2). Univariate analysis carried forward in the population of patients as a whole and adjusted for dialysis vintage, identified PTX as a protective factor for overall survival (Table 3; *p* = 0.002). Similarly, higher serum albumin (*p* = 0.0001), higher hemoglobin levels (*p* = 0.0001) and younger age (*p* = 0.0001) were associated with better survival. As reported in Table 3, multivariable analysis confirmed PTX as an independent factor of better survival while age and dialysis vintage were associated with worse outcome. On a subset of propensity-score matched patients, well balanced for sex, age, dialysis vintage, diabetes, albumin, hemoglobin, Calcium, Phospahte, PTH and therapies (Table 4), PTX was confirmed to be a protective factor for overall survival (HR: 0.404 [0.254–0.643]; *p* = 0.00132).

**Table 2:** Prevalence of drug prescriptions during follow-up

	PTX	Non-PTX	*P*
Baseline			
Total patients	257	418	
Phosphate binders, %	66	77	0.002
Calcium binders, %	66	36	< .0001
Non calcium based binders, %	85	99	< .0001
Vitamin D, %	41	65	< .0001
Calcitriol (p.o + e.v), %	58	44	0.0039 *
Paricalcitol, %	44	68	0.0384*
Other, %	12	18	0.1313*
Cinacalcet, %	14	34	< .0001
First-year follow-up			
Total patients	221	295	
Phosphate binders, %	75	89	< .0001
Calcium binders, %	67	41	0.001
Non calcium based binders, %	80	87	0.001
Vitamin D, %	52	68	< .0001
Calcitriol (p.o + e.v), %	57	30	< .0001*
Paricalcitol, %	43	62	0.036*
Other, %	11	25	0.027*
Cinacalcet, %	22	39	< .0001
Second-year follow-up			
Total patients	183	230	
Phosphate binders, %	83	88	0.001
Calcium based binders, %	68	35	< 0.0001
Non calcium based binders, %	77	84	< 0.0001
Vitamin D, %	61	77	0.0006
Calcitriol (p.o + e.v), %	57	25	< .0001*
Paricalcitol, %	48	67	0.018 *
Other, %	16	29	0.060*
Cinacalcet, %	16	41	< .0001
Third-year follow-up			
Total patients	132	144	
Phosphate binders, %	71	84	0.014
Calcium based binders, %	62	33	0.0143
Non calcium based binders, %	91	92	0.0143
Vitamin D, %	50	80	< .0001
Calcitriol (p.o + e.v), %	61	21	< .0001*
Paricalcitol, %	28	75	< .0001 *
Other, %	36	27	0.9684*
Cinacalcet, %	19	39	0.0004

Data are shown as percentage. Chi-squared test was used to test for significant differences between groups

^*^Bonferroni *p*-adjusted

Abbreviations: *PTX* parathyroidectomy

**Table 3:** Univariate and multivariate survival analyses

Univariate analysis				
Variable	HR	CI, Low	CI, Up	p
PTX	0.556	0.387	0.800	0.002
Age, years	1.047	1.032	1.062	0.0001
Gender (Male)	0.799	0.572	1.116	0.189
Ca, mg/dl	1.269	0.974	1.654	0.078
Phosphate, mg/dl	0.950	0.891	1.100	0.491
PTH, pg/ml	1.000	0.999	1.001	0.966
Albumin, g/dl	0.372	0.249	0.555	0.0001
Hb, g/dl	0.672	0.558	0.810	0.0001
Multivariate analysis				
PTX	0.6719	0.4652	0.9706	0.034
Dialysis vintage, years	1.0345	1.0143	1.0551	0.0001
Age, years	1.0438	1.0286	1.0592	0.0001

Abbreviations: *PTX* Parathyroidectomy; *Ca* calcium; *P* phosphate; *PTH* parathyroid hormone; *Hb* hemoglobin; *CI* confidence interval

**Table 4:** Balance measures pre- and post- matching

Variable	Pre–matching SMD	Post–matching SMD
Age, years	– 0.1734	0.0432
Gender (Male)	– 0.0012	– 0.0748
Dialysis vintage, years	– 0.4237	– 0.0394
Diabetes	– 0.2985	– 0.0610
Albumin, g/dl	0.3369	0.0074
Hb, g/dl	0.0619	– 0.0705
Ca, mg/dl	– 0.3366	0.0531
Phosphate, mg/dl	– 0.1489	– 0.0419
PTH, pg/ml	– 0.5105	0.0659
Cinacalcet	– 0.5822	0.0254
Phosphate binders		
Calcium based binders	0.5411	0.0841
Non calcium based binders	– 0.2777	0.0489
Vitamin D		
Calcitriol	0.5179	– 0.0111
Paricalcitol	– 0.8194	– 0.0066
Other	– 0.4859	0.0262

Threshold for well-balanced variable: > .1

Abbreviations *SMD* standardized mean difference; *Hb* hemoglobin; *Ca* calcium; *P* phosphate; *PTH* parathyroid hormone

## Discussion

The main result of our study is that PTX was associated with a better survival rate in our HD population of 257 PTX patients compared with 418 matched non-PTX patients, prospectively followed-up for three years. This result was confirmed in both univariable and multivariable-adjusted survival analyses (Table 3). Interestingly, the percentage of patients with adequate calcium and phosphate control did not differ between the PTX and non-PTX groups, while PTH levels were less frequently at target during the three years of follow-up in the PTX group (Fig. 4). In particular, during the three years of follow-up, the PTX group was mostly and invariably exposed to very low PTH levels. In our study, given the time frame, we used the KDOQI target ranges. As a comparison, we also used the more recent KDIGO PTH targets, which confirmed the significant differences between PTX and non-PTX patients (below target: 65 vs 24%, *p* < 0.0001; at target 30 vs 66%, *p* < 0.0001; above target 5 vs 10%, *p* < 0.01. Supplemental material, Fig. 2). Therefore, the association between PTX and a better overall survival rate appears to be independent of the attained biochemical profile. Notably, the similar biochemical control of calcium, phosphate and PTH resulted from a lower prescription of active vitamin D, phosphate binders and calcimimetics in the PTX group (Fig. 5), thus pointing to the role of still poorly known but commonly recognized limitations of the widely employed therapeutic strategies for SHPT. The Kaplan–Meier survival analysis showed better survival rates of the PTX group, in particular in the long term (Fig. 2). Indeed, the PTX group had undergone surgery on average 8 years after hemodialysis inception and the survival curves progressively diverged over time, remaining significant even after 30 years of follow-up. It is also interesting to notice that although multivariable analysis identified dialysis vintage as a risk factor of mortality, the PTX group had better survival, despite a longer dialysis vintage. Overall, our data suggest that PTX has a long-term protective effect on survival in HD patients. Our results are in agreement with the available evidence in the literature showing reduced all-cause and cardiovascular mortality in the long term after PTX [13–15, 21], again independently of the biochemical control of mineral metabolism. We can therefore look back at the concept of PTH as a uremic toxin [23]. In fact, we are aware that PTH may have several extra-mineral negative effects in dialysis patients, spanning from increased left ventricular hypertrophy and higher blood pressure to erythropoietin-resistant anemia and poor nutrition and quality of life [24–26]. In our opinion, it is possible that PTX, by shortening exposure to high PTH levels, reduces the effects of extra-mineral damage.

The main strength of our paper is the multicenter, observational, prospective study design which allows the evaluation of real-life therapeutic strategies. On the other hand, we acknowledge that the enrollment of prevalent PTX hemodialysis patients that underwent surgery during their dialytic history represents a limitation. In fact, enrolling prevalent instead of incident PTX patients carries a number of potential selection biases, as clearly reported in the literature [22]. However, randomized controlled trials comparing patients receiving surgical or medical therapy at the time of surgical indication for severe SHPT do not exist and, most likely, will never be carried out [22]. In conclusion, PTX can be regarded as an effective and safe therapy for refractory SHPT in dialysis patients even though the metabolic control reached after surgery may not be optimal.

### Supplementary Information

Below is the link to the electronic supplementary material.

Supplementary file1 (PDF 411 kb)
