# Supplementary material for: Parathyroidectomy and survival in a cohort of Italian dialysis patients: results of a multicenter, observational, prospective study
Source: J Nephrol. 2023 Jun 23;36(7):1947–55. doi: 10.1007/s40620-023-01658-0 (PMC10543527; doi:10.1007/s40620-023-01658-0)
Supplement: Supplementary file 1 — Supplementary file1 (PDF 411 kb) [file 40620_2023_1658_MOESM1_ESM.pdf]

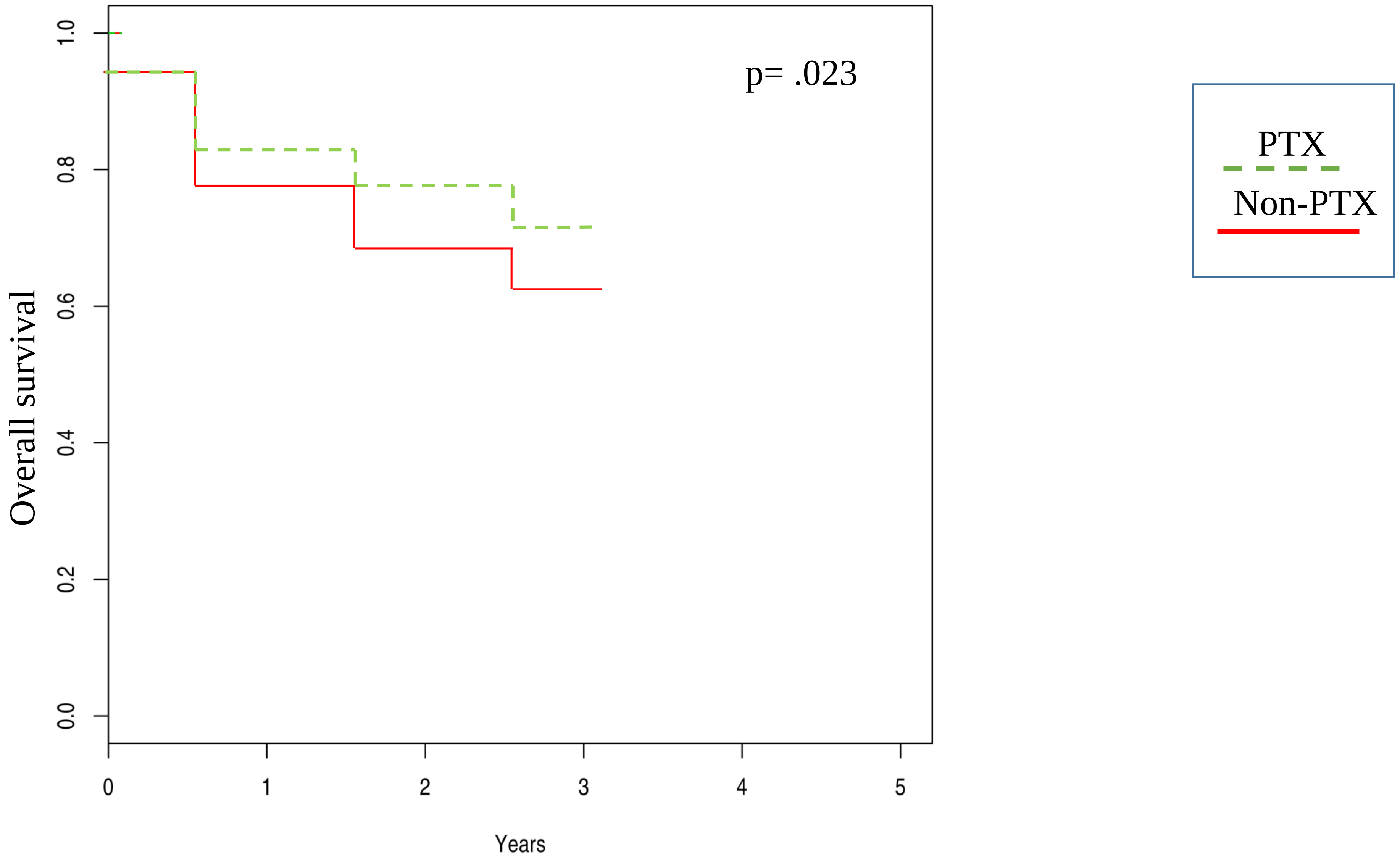

Time, years

Figure 1 s Overall survival, PTX vs. control group. Time 0 was the date of starting enrolment. Kaplan-Meier log rank test = 0.023

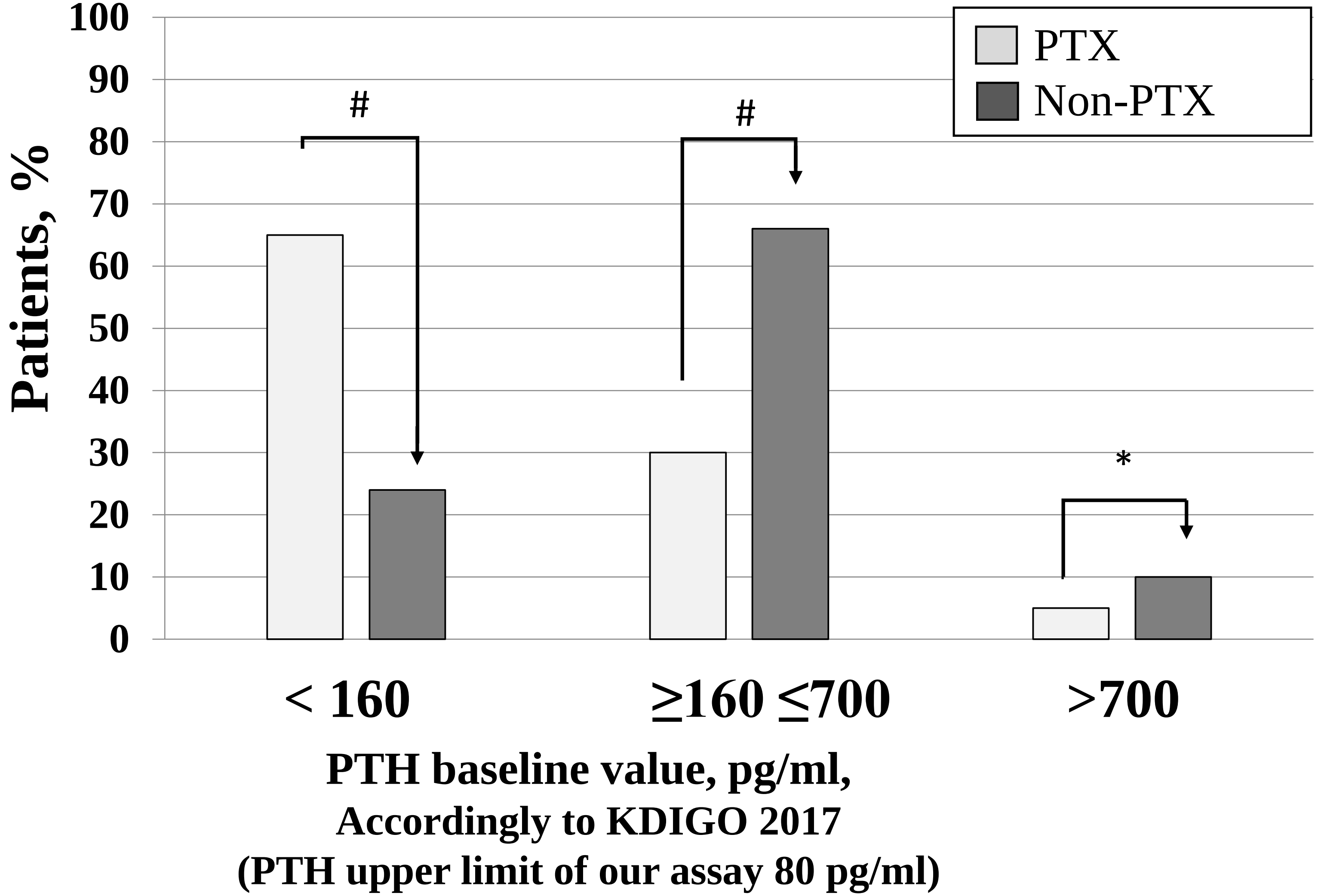

Figure 2 s. Percentage of patients with low (<160 pg/ml), at target (≥160 ≤ 700, pg/ml) and high (>700 pg/ml) PTH value at baseline (year 2011). Accordingly to KDIGO 2017 guidelines PTH target value are 2 to 9 times the upper normal limit for the assay (upper limit 80 pg/ml)

#PTX vs C X^2, p= 0.0001;* PTX vs C X^2, p= 0.01
